# Low-contrast lesion detection in neck CT: a multireader study comparing deep learning, iterative, and filtered back projection reconstructions using realistic phantoms

**DOI:** 10.1186/s41747-024-00486-6

**Published:** 2024-07-24

**Authors:** Quirin Bellmann, Yang Peng, Ulrich Genske, Li Yan, Moritz Wagner, Paul Jahnke

**Affiliations:** 1 Department of Radiology, Charité—Universitätsmedizin Berlin, Corporate Member of Freie Universität Berlin, Humboldt-Universität zu Berlin, and Berlin Institute of Health, Charitéplatz 1, 10117 Berlin, Germany; 2 Department of Radiology, Tongji Hospital, Tongji Medical College, Huazhong University of Science and Technology, 1095 Jiefang Avenue, Wuhan, 430030 Hubei Province China; 3 Berlin Institute of Health (BIH), Anna-Louisa-Karsch-Str. 2, 10178 Berlin, Germany

**Keywords:** Deep learning, Neck, Parapharyngeal space, Phantoms (imaging), Tomography (x-ray computed)

## Abstract

**Background:**

Computed tomography (CT) reconstruction algorithms can improve image quality, especially deep learning reconstruction (DLR). We compared DLR, iterative reconstruction (IR), and filtered back projection (FBP) for lesion detection in neck CT.

**Methods:**

Nine patient-mimicking neck phantoms were examined with a 320-slice scanner at six doses: 0.5, 1, 1.6, 2.1, 3.1, and 5.2 mGy. Each of eight phantoms contained one circular lesion (diameter 1 cm; contrast -30 HU to the background) in the parapharyngeal space; one phantom had no lesions. Reconstruction was made using FBP, IR, and DLR. Thirteen readers were tasked with identifying and localizing lesions in 32 images with a lesion and 20 without lesions for each dose and reconstruction algorithm. Receiver operating characteristic (ROC) and localization ROC (LROC) analysis were performed.

**Results:**

DLR improved lesion detection with ROC area under the curve (AUC) 0.724 ± 0.023 (mean ± standard error of the mean) using DLR *versus* 0.696 ± 0.021 using IR (*p* = 0.037) and 0.671 ± 0.023 using FBP (*p* < 0.001). Likewise, DLR improved lesion localization, with LROC AUC 0.407 ± 0.039 *versus* 0.338 ± 0.041 using IR (*p* = 0.002) and 0.313 ± 0.044 using FBP (*p* < 0.001). Dose reduction to 0.5 mGy compromised lesion detection in FBP-reconstructed images compared to doses ≥ 2.1 mGy (*p* ≤ 0.024), while no effect was observed with DLR or IR (*p* ≥ 0.058).

**Conclusion:**

DLR improved the detectability of lesions in neck CT imaging. Dose reduction to 0.5 mGy maintained lesion detectability when denoising reconstruction was used.

**Relevance statement:**

Deep learning enhances lesion detection in neck CT imaging compared to iterative reconstruction and filtered back projection, offering improved diagnostic performance and potential for x-ray dose reduction.

**Key Points:**

- Low-contrast lesion detectability was assessed in anatomically realistic neck CT phantoms.
- Deep learning reconstruction (DLR) outperformed filtered back projection and iterative reconstruction.
- Dose has little impact on lesion detectability against anatomical background structures.

**Graphical Abstract:**

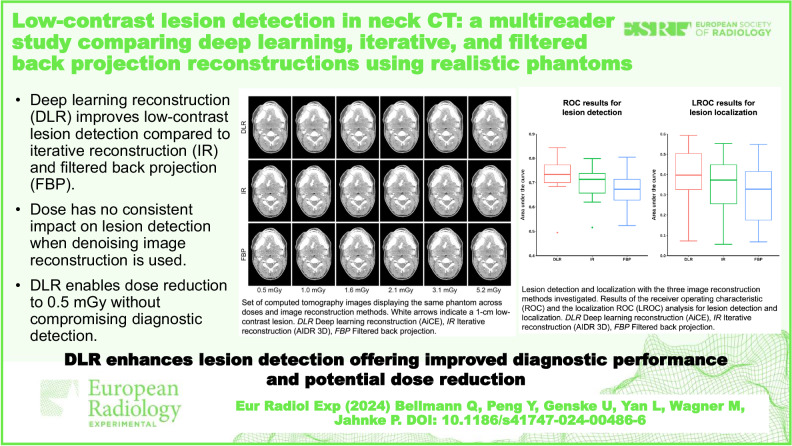

## Background

Image reconstruction algorithms in computed tomography (CT) improve image quality and dose efficiency by optimizing raw data processing and photon yield. In modern CT scanners, iterative reconstruction (IR) methods, with their strong denoising capabilities, have largely replaced traditional filtered back projection (FBP) methods. More recently, the latest generation of deep learning reconstruction (DLR) algorithms has been introduced to address the limitations of IR and to further optimize photon yield [1].

IR methods use nonlinear operations to denoise images and can maintain an acceptable contrast-to-noise ratio even at very low x-ray doses [2]. However, IR also alters image texture and affects contrast-dependent spatial resolution, which in turn may degrade lesion detectability and diagnostic confidence [3]. In contrast, DLR methods using convolutional neural networks have been reported to denoise images without introducing alterations in noise texture commonly associated with IR [4, 5]. DLR may therefore enable more reliable lesion detection and improve diagnostic performance.

Several phantom studies indicate superior low-contrast detection performance for images reconstructed using DLR compared with IR [6, 7]. However, these studies were conducted on uniform phantoms, and it has been shown that the complexity of background texture significantly affects low-contrast lesion detection tasks [8, 9]. Only a few studies have addressed the potential of DLR to actually improve lesion detection in patients, and thus far, the emphasis has been on abdominal imaging [10, 11]. Performing this type of evaluation in patient studies faces challenges including limited patient availability, dose exposure concerns, difficulties in reproducibility, and a lack of ground truth knowledge, which is essential to validate detection outcomes.

To address these challenges, previous work has presented realistic neck phantoms, which allow researchers to combine the advantages of studying low-contrast detectability in patients (offering realism) and phantoms (ensuring standardization) [12]. In an assessment of these phantoms, radiologists found lesions of 1 cm in diameter and -30 HU contrast to the background to be at the threshold of detectability.

In the present study, we used this type of phantom to evaluate lesion detectability by comparing DLR, IR, and FBP at six doses. The study was motivated by the hypothesis that DLR improves lesion detection in anatomical backgrounds. Based on this assumption, the aim of the study was to evaluate DLR for low-contrast lesion detection in neck CT imaging in comparison with IR and FBP.

## Methods

### Study design

The institutional Ethics Committee approved the study (see Declarations) and waived informed consent. Nine anatomically realistic neck phantoms were examined by CT with six different radiation doses (each of eight phantoms containing a low-contrast lesion and one phantom not containing any lesion). Images were reconstructed using DLR, IR, and FBP. Lesion detectability was evaluated by 13 radiologists.

### Phantoms

**Fig. 1:**
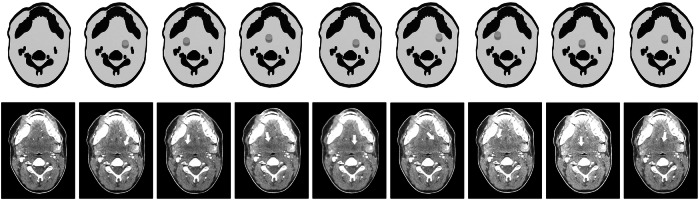
Drawings and computed tomography images of the phantoms. Cylindrical lesions are drawn in gray and indicated by white arrows in the images. Images were acquired with a tube current of 100 mA and reconstructed with the manufacturer’s implementation of deep learning reconstruction (AiCE)

The design, production, and validation of the phantoms used in this study have been reported in detail in previous work [12]. Briefly, circular lesions of 1 cm in diameter and -30 HU contrast were digitally inserted at eight different positions in the parapharyngeal space into a contrast-enhanced neck CT image of a female patient aged 22 years who had undergone the examination following a traffic accident (lesions were inserted by pixel-wise subtraction of 30 HU). The selected lesion contrast aimed to position the lesions at the interface between detectable and undetectable, as determined by earlier research [12, 13]. The original non-lesion image and the eight lesion-containing images were then used to create nine phantoms of 1-cm thickness using radiopaque three-dimensional printing [14, 15]. The resulting phantoms each contained the same anatomy and the same lesion (or no lesion) across the entire thickness of 1 cm. They differed only in lesion position or absence, but not in anatomical background. Figure 1 shows CT scans of each phantom and illustrates lesion positions.

### Image acquisition

The phantoms were scanned using a Canon Aquilion One Genesis CT scanner (Canon Medical Systems, Otawara, Japan). The tube voltage was 120 kVp, the rotation time 0.5 s, the pitch was 0.813, the field of view had a diameter of 280 mm, and the image matrix was 512 × 512 pixels. Fixed tube currents of 10, 20, 30, 40, 60, and 100 mA were used, corresponding to volume CT dose indices−CTDI_vol_ of 0.5, 1, 1.6, 2.1, 3.1, and 5.2 mGy. Five acquisitions were performed per dose and tube current. Images were reconstructed with 1-mm slice thickness and 0.8-mm increment using FBP with a soft tissue kernel (FC08) and the manufacturer’s implementation of IR and DLR: Adaptive Iterative Dose Reduction 3D (AIDR 3D) and Advanced intelligent Clear-IQ Engine (AiCE). One central image slice per acquisition and reconstruction of the lesion phantoms and four central slices per acquisition and reconstruction of the non-lesion phantom were extracted for the subsequent reading experiment.

### Lesion detectability assessment

Thirteen observers participated in a reading experiment to evaluate low-contrast lesion detectability in the phantoms. Six participants were board-certified radiologists, seven participants were radiologists in training. Reader experience in neck CT imaging ranged from 3 to 14 years (median 4 years). For every dose and image reconstruction method, readers were presented with 32 images of the lesion phantoms (4 images per phantom) and 20 images of the non-lesion phantom. The experiment thus encompassed 936 images per reader (6 doses × 3 reconstruction methods × 52 images). Images were presented individually. Readers were asked to decide whether images contained a lesion in the parapharyngeal space and to indicate their confidence on a seven-point scale (1 = definitely absent; 2 = probably/possibly absent; 3 = unsure of lesion absence or presence; 4 = probably/possibly present; 5 = definitely present). In addition, they were asked to label lesions when deemed present by placing a circular region of interest (ROI). ROIs were adjustable, enabling readers to label lesions exactly as they observed them. Participants were instructed to search for a maximum of one circular low-contrast lesion of 1 cm in diameter per image. Every reader completed a training session involving 20 images at 5.2 mGy prior to the experiment to get familiar with the experimental setup, including the process of labeling ROIs. Readings were randomly assigned and readers were unaware of lesion positions and the number of possible different lesion positions, forcing them to perform a search task for each presented image. No consensus agreement was made. Readings were performed in four separate sessions; the interval between reading sessions ranged from 1 to 58 days (median 1 day). There was no time limit, enabling readers to pause in case of fatigue. Images were read on diagnostic workstations using a dedicated open-source software platform (Human Observer Net) [16].

### Statistical analysis

To analyze reader responses to lesion absence or presence, the data was formatted and analyzed according to the receiver operating characteristic (ROC) paradigm using only the confidence scores of the readings as previously described [17, 18]. Briefly, reader responses to lesion absence or presence were used to calculate the true positive fraction and the false positive fraction for each reader at different decision thresholds. True-positive reader responses occurred when readers correctly identified images of lesion phantoms as lesion images, whereas false-positive responses occurred when readers incorrectly identified images of the non-lesion phantom as lesion images. These results were subsequently used to create ROC curves from which area under the curve (AUC) values were derived. For the analysis of lesion localization, the Dice similarity coefficient (DSC) was calculated for each image in which readers outlined a lesion [19, 20]. The DSC was used to calculate the overlap between ROIs placed by readers and the ground truth ROI. Ground truth ROIs were determined during the study setup in Human Observer Net [16] by the position, size, and shape of lesion insertions used for phantom production, defining the phantom ground truth. A DSC ≥ 0.5 (corresponding to ≥ 50% overlap) was used as the threshold to classify reader responses as correct lesion identification. The DSC results and confidence scores were analyzed following the localization ROC (LROC) paradigm as described in [17, 18]. Briefly, the true positive fraction and false positive fraction were calculated based on the combination of the DSC and confidence scores at different decision thresholds, which means that reader responses were only counted as true positives if the DSC was ≥ 0.5. True positive fraction and false positive fraction results were used to create LROC curves and calculate associated AUC values for each reader. Statistical analysis of the AUC values derived from the ROC and LROC datasets was performed according to the Dorfman-Berbaum-Metz method [17, 18]. Readers were treated as a random factor while cases were considered fixed. AUC values resulting from the ROC and LROC analysis were compared among image reconstruction methods. In addition, a subanalysis was performed to evaluate dose effects for each image reconstruction method. Bonferroni correction was applied to adjust *p*-values for multiple comparisons. In another subanalysis, lesion detection, and localization were analyzed according to reader experience. To this end, readers were divided into two groups: (i) 7 radiologists in training with 3 to 4 years of experience; and (ii) 6 board-certified radiologists with 6 to 14 years’ experience. For each reader, ROC and LROC curves and associated AUC values were calculated using all confidence ratings and lesion localizations. An unpaired Student *t*-test was applied to compare the AUC values of the two reader groups. Differences were interpreted as significant for *p* < 0.05. Data was processed using R (v4.3.2). The tidyverse (v2.0.0) collection of R packages was used for data preprocessing and plotting. For statistical analysis, the R packages RJafroc (v2.1.2) and ggpubr (v0.6.0) were utilized.

## Results

### Effects of image reconstruction method

**Fig. 2:**
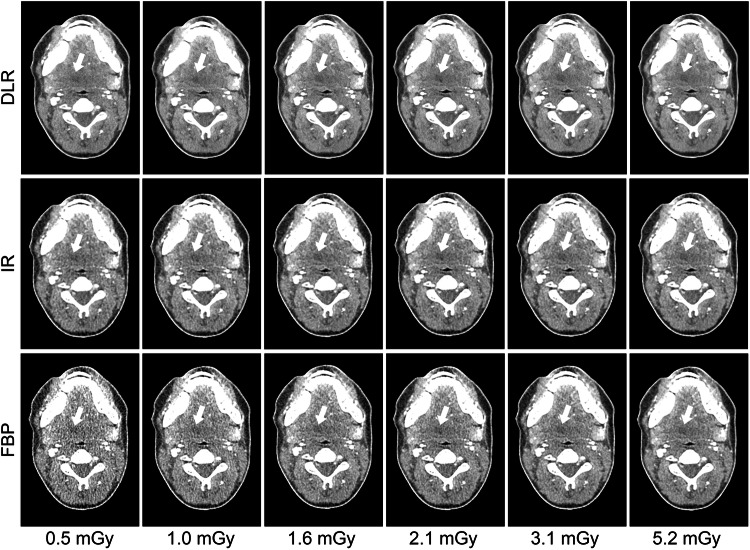
Set of computed tomography images across doses and image reconstruction methods. All images are displayed with 40/350 HU window level/window width. DLR, Deep learning reconstruction (AiCE); IR, Iterative reconstruction (AIDR 3D); FBP, Filtered back projection

**Fig. 3:**
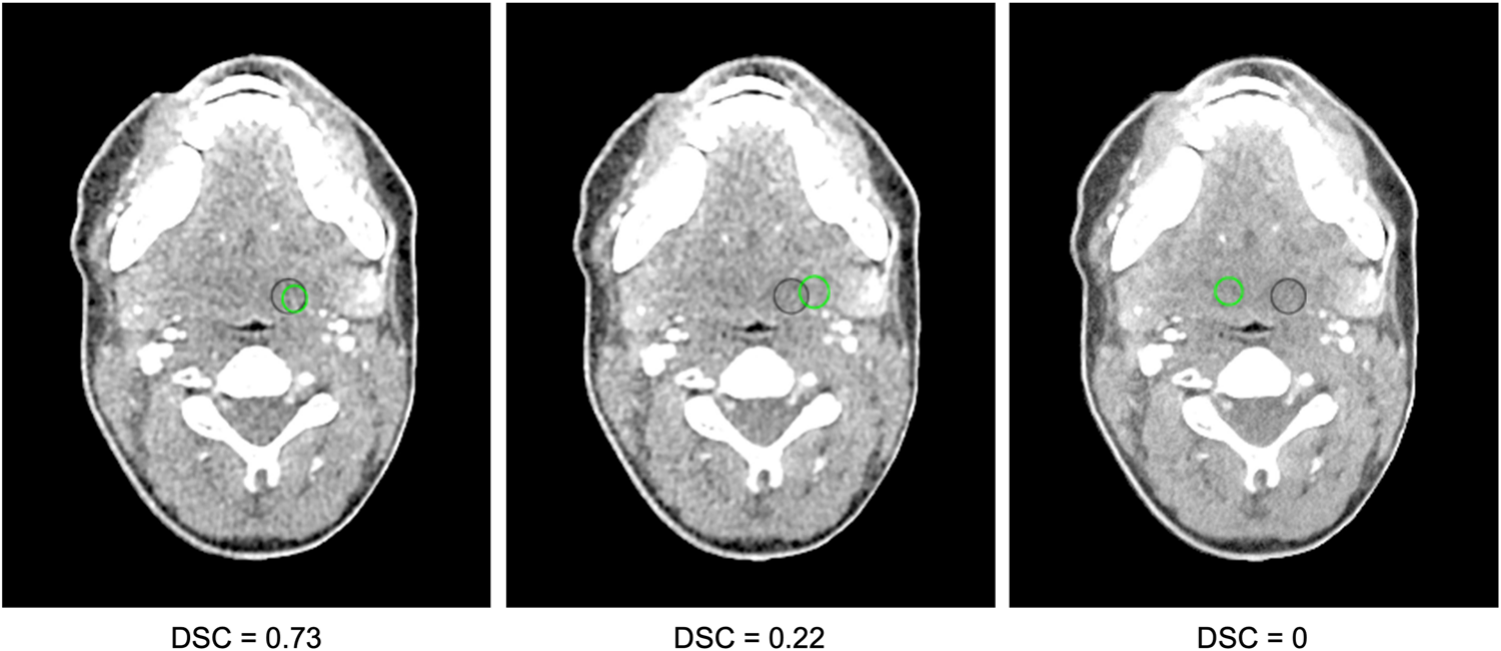
Set of computed tomography images demonstrating lesion labeling by study participants. The lesion ground truth in the left parapharyngeal space is indicated by a black region of interest (ROI). ROIs placed by readers for lesion labeling are indicated in green. Left: the Dice similarity coefficient (DSC) indicating the overlap between the ROI placed by the reader and the ground truth ROI was ≥ 0.5. Consequently, the reader response was classified as correct lesion identification. Middle and right: The DSC was < 0.5, and reader responses were thus classified as incorrect

**Fig. 4:**
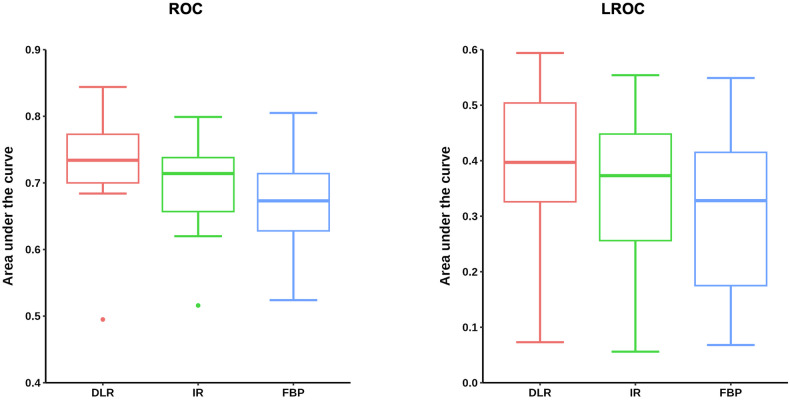
Lesion detection and localization with the three image reconstruction methods investigated. Results of the receiver operating characteristic (ROC) and the localization ROC (LROC) analysis for lesion detection and localization. DLR, Deep learning reconstruction (AiCE); IR, Iterative reconstruction (AIDR 3D); FBP, Filtered back projection

Images reconstructed with DLR, IR, and FBP across all six doses investigated in this study are shown in Fig. 2. Figure 3 presents a set of CT images demonstrating lesion labels placed by participants. AUC results by reconstruction method are presented in Fig. 4. DLR improved reader performance and confidence in detecting lesion images compared with IR (*p* = 0.037) and FBP (*p* < 0.001). The mean ± standard error of the mean (SEM) AUC obtained by the ROC analysis was 0.724 ± 0.023 for DLR *versus* 0.696 ± 0.021 for IR and 0.671 ± 0.023 for FBP. IR did not yield significantly better results than FBP (*p* = 0.057). The superiority of DLR was further confirmed by the LROC analysis, showing that greater reader confidence was associated with improved lesion delineation. The mean ± SEM AUC resulting from the LROC analysis was 0.407 ± 0.039 for DLR, compared with 0.338 ± 0.041 for IR (*p* = 0.002) and 0.313 ± 0.044 for FBP (*p* < 0.001). There was no statistically significant difference between IR and FBP in the LROC analysis (*p* ≥ 0.423).

### Effects of dose

**Fig. 5:**
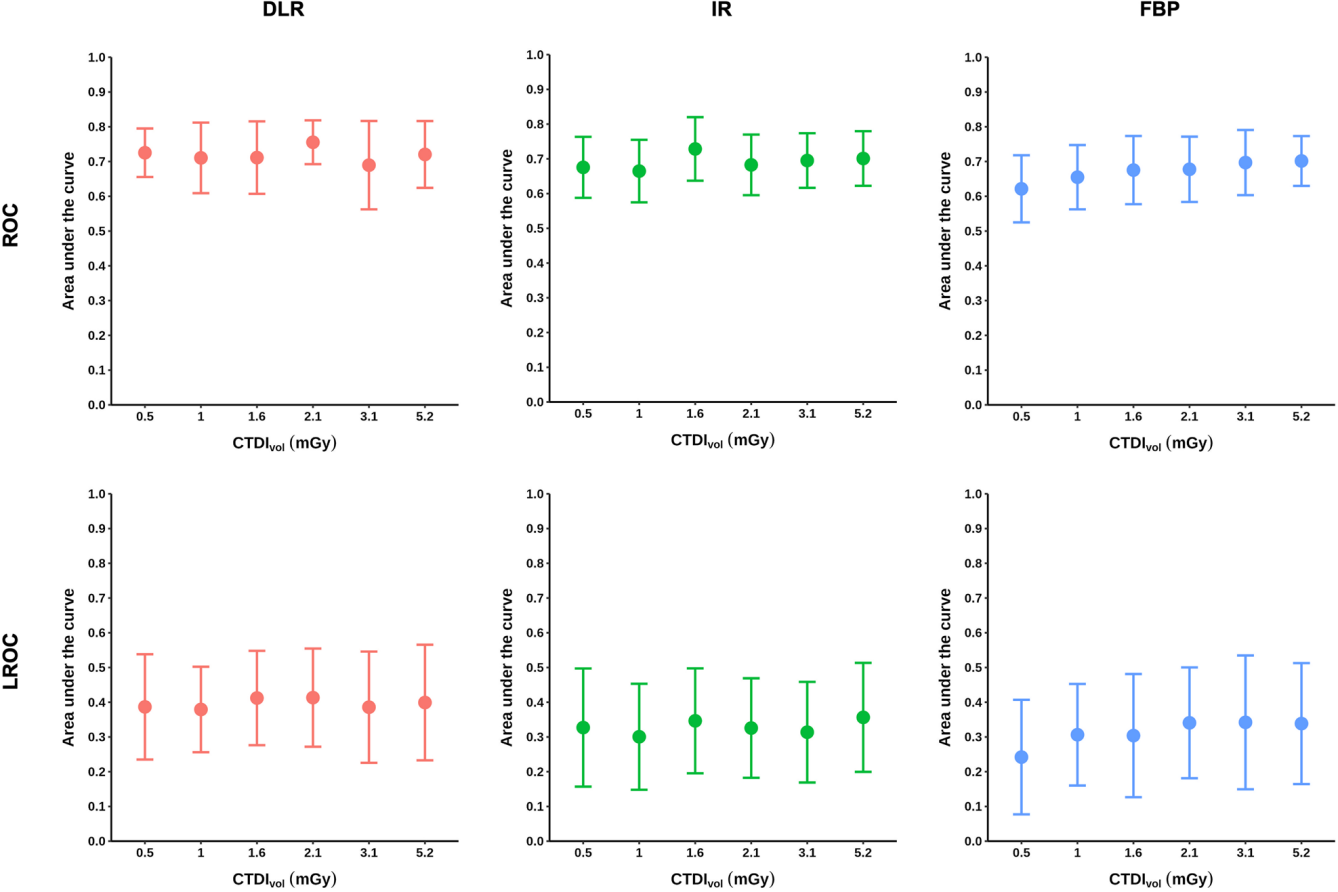
Lesion detection and localization by dose and image reconstruction method. Averaged results of the receiver operating characteristic (ROC) and the localization ROC (LROC) analysis for lesion detection and localization. Error bars indicate standard deviations. DLR, Deep learning reconstruction (AiCE); IR*,* Iterative reconstruction (AIDR 3D); FBP*,* Filtered back projection; CTDIvol*,* Volume computed tomography dose index

Figure 5 shows AUC results per dose and image reconstruction method. Numerical results are provided in Tables 1 and 2. Tables 3 and 4 present *p*-values resulting from dose comparisons. Dose reduction to 0.5 mGy significantly compromised readers’ ability to correctly identify FBP-reconstructed lesion images compared to 1.6, 2.1, 3.1, and 5.2 mGy. Likewise, dose reduction to 0.5 mGy compromised lesion localization in FBP-reconstructed images compared to 2.1, 3.1, and 5.2 mGy. In contrast, no significant dose effects were observed when DLR or IR was used for image reconstruction, except for ROC results at 1.6 mGy with IR, which were superior to those at 1 mGy and also showed an increase compared to 0.5 mGy, though without reaching statistical significance. However, unlike FBP, these observations were incidental, as no other dose comparisons using IR or DLR yielded consistent effects. Moreover, these observations were not confirmed by the LROC analysis, which showed no significant dose effects in images reconstructed with IR or DLR at any dose. There was a trend toward higher detection as the dose increased in FBP-reconstructed images, whereas no consistent trend was observed with DLR or IR.

**Table 1:** Results of the receiver operating characteristic (ROC) analysis by dose and image reconstruction method

CTDIvol (mGy)	DLR	IR	FBP
0.5	0.725 ± 0.019	0.675 ± 0.024	0.621 ± 0.027
1.0	0.711 ± 0.028	0.665 ± 0.025	0.655 ± 0.025
1.6	0.711 ± 0.029	0.729 ± 0.025	0.675 ± 0.027
2.1	0.755 ± 0.017	0.683 ± 0.024	0.678 ± 0.026
3.1	0.690 ± 0.035	0.695 ± 0.022	0.697 ± 0.026
5.2	0.720 ± 0.027	0.701 ± 0.022	0.702 ± 0.020

Data are presented as mean ± standard error of the mean area under the curve

*DLR* Deep learning reconstruction (AiCE), *IR* Iterative reconstruction (AIDR 3D), *FBP* Filtered back projection, *CTDIvol* Volume computed tomography dose index

**Table 2:** Results of the localization receiver operating characteristic (LROC) analysis by dose and image reconstruction method

CTDIvol (mGy)	DLR	IR	FBP
0.5	0.387 ± 0.042	0.327 ± 0.047	0.242 ± 0.045
1.0	0.379 ± 0.034	0.301 ± 0.042	0.306 ± 0.041
1.6	0.412 ± 0.038	0.346 ± 0.041	0.304 ± 0.044
2.1	0.413 ± 0.039	0.326 ± 0.040	0.341 ± 0.044
3.1	0.386 ± 0.044	0.314 ± 0.040	0.342 ± 0.053
5.2	0.399 ± 0.046	0.356 ± 0.044	0.339 ± 0.048

Data are presented as mean ± standard error of the mean area under the curve

*DLR* Deep learning reconstruction (AiCE), *IR* Iterative reconstruction (AIDR 3D), *FBP* Filtered back projection, *CTDIvol* Volume computed tomography dose index

**Table 3:** Comparison of the receiver operating characteristic (ROC) results by dose

CTDIvol (mGy)	DLR	IR	FBP
0.5 *versus* 1.0	1	1	0.806
0.5 *versus* 1.6	1	0.058	0.035
0.5 *versus* 2.1	1	1	0.024
0.5 *versus* 3.1	1	1	< 0.001
0.5 *versus* 5.2	1	1	< 0.001
1.0 *versus* 1.6	1	0.009	1
1.0 *versus* 2.1	0.789	1	1
1.0 *versus* 3.1	1	1	0.245
1.0 *versus* 5.2	1	0.667	0.119
1.6 *versus* 2.1	0.846	0.178	1
1.6 *versus* 3.1	1	1	1
1.6 *versus* 5.2	1	1	1
2.1 *versus* 3.1	0.078	1	1
2.1 *versus* 5.2	1	1	1
3.1 *versus* 5.2	1	1	1

*p*-values are presented

*DLR* Deep learning reconstruction (AiCE), *IR* Iterative reconstruction (AIDR 3D), *FBP* Filtered back projection, *CTDIvol* Volume computed tomography dose index

**Table 4:** Comparison of the localization receiver operating characteristic (LROC) results by dose

CTDIvol (mGy)	DLR	IR	FBP
0.5 *versus* 1.0	1	1	0.257
0.5 *versus* 1.6	1	1	0.329
0.5 *versus* 2.1	1	1	0.006
0.5 *versus* 3.1	1	1	0.005
0.5 *versus* 5.2	1	1	0.008
1.0 *versus* 1.6	1	0.658	1
1.0 *versus* 2.1	1	1	1
1.0 *versus* 3.1	1	1	1
1.0 *versus* 5.2	1	0.222	1
1.6 *versus* 2.1	1	1	1
1.6 *versus* 3.1	1	1	1
1.6 *versus* 5.2	1	1	1
2.1 *versus* 3.1	1	1	1
2.1 *versus* 5.2	1	1	1
3.1 *versus* 5.2	1	1	1

*p*-values are presented

*DLR* Deep learning reconstruction (AiCE), *IR* Iterative reconstruction (AIDR 3D), *FBP* Filtered back projection, *CTDIvol* Volume computed tomography dose index

### Reader experience

**Fig. 6:**
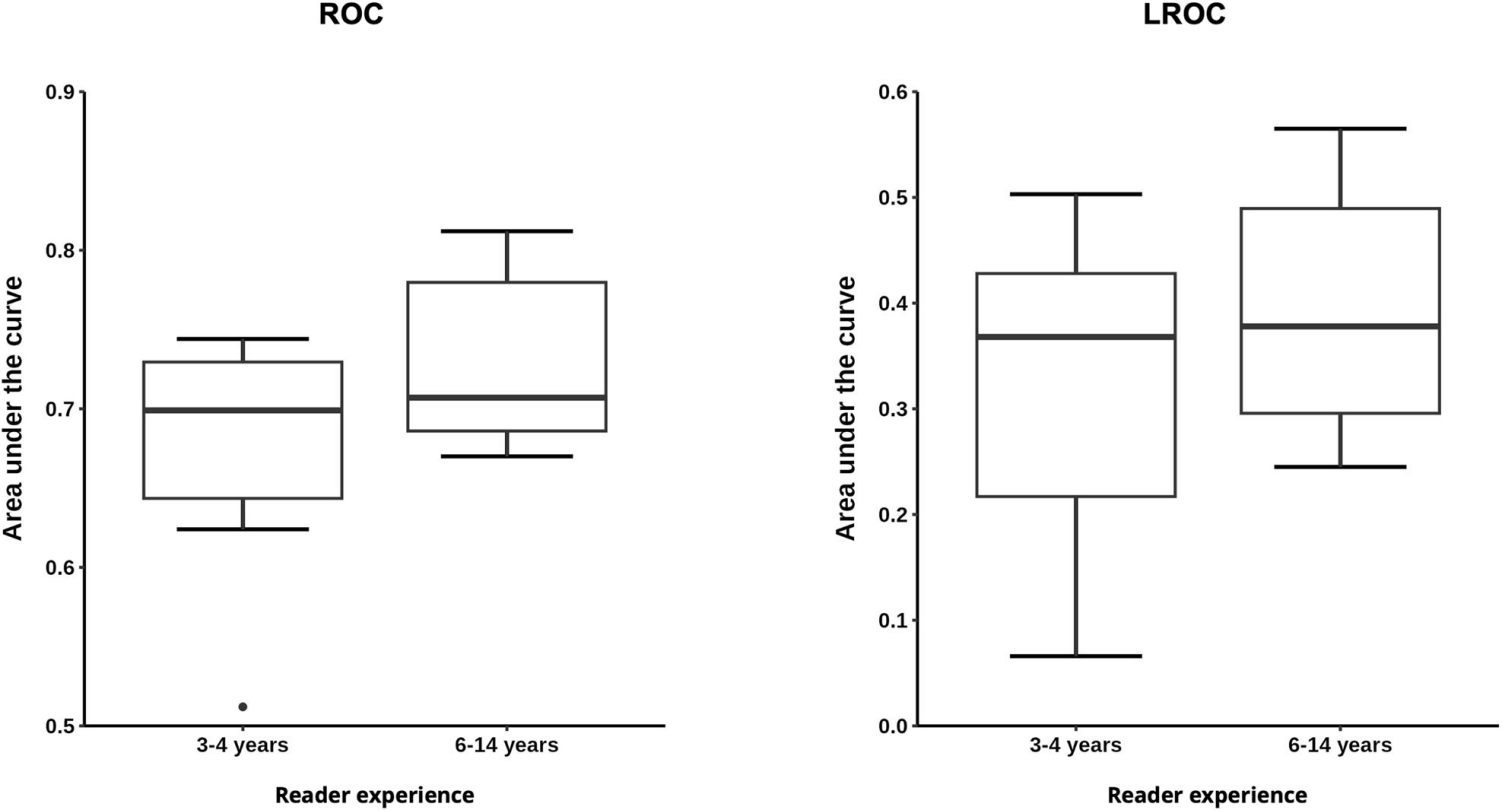
Comparison of lesion detection and localization by reader experience. Results of the receiver operating characteristic (ROC) and the localization ROC (LROC) analysis for lesion detection and localization grouped by reader experience of 3–4 years (7 participants) and 6–14 years (6 participants)

Figure 6 shows AUC results from the subanalysis of reader experience. The mean ± SEM AUC obtained from the ROC analysis was 0.73 ± 0.024 for the more experienced reader group (6 to 14 years of experience) *versus* 0.672 ± 0.029 for the less experienced group (3 to 4 years of experience). The difference between these groups was not statistically significant (*p* = 0.173). Likewise, the LROC analysis yielded slightly superior AUC results in the more experienced group without reaching statistical significance. The mean ± SEM AUC resulting from the LROC analysis was 0.394 ± 0.053 for the more experienced group *versus* 0.318 ± 0.059 for the less experienced group (*p* = 0.364).

## Discussion

This multi-reader study, conducted with nine anthropomorphic phantoms, revealed that DLR improves the detectability of low-contrast lesions in CT imaging of the neck compared with IR and FBP across doses from 0.5 to 5.2 mGy (*p* ≤ 0.037). Dose reduction to 0.5 mGy impaired lesion detection in FBP-reconstructed images compared with doses ≥ 2.1 mGy (*p* ≤ 0.024), but had no significant impact when DLR or IR was used.

Lower image noise aids radiologists in distinguishing signals from noise and explains why IR yielded better detection results than FBP in previous work [21, 22]. However, other studies reported only minor or no significant advantages of using IR [23–25]. Our findings align with these observations, demonstrating only slightly improved detection compared to FBP, which did not reach statistical significance. This constraint on improvement from IR can be explained by texture shifts that result in low-frequency noise, which can adversely impact the detectability of lesions [3]. Newer DLR methods have been reported to no longer exhibit such changes in noise frequency, suggesting their potential for a more favorable noise texture. Our results confirm that DLR further improves lesion detectability, thus supporting prior reports of improved denoising performance compared with IR [5].

We found moderate dose effects in FBP-reconstructed images and no consistent effects when IR or DLR was used. In FBP, dose is inversely correlated with image noise, and excessive noise at low doses could be expected to obscure signals and impair lesion detection. This assumption was to some extent confirmed by the marked decrease in lesion detection we observed at the lowest dose of 0.5 mGy. Overall, however, dose effects were less pronounced than expected. Moreover, the application of denoising image reconstruction showed no consistent impact from dose modifications, as improved ROC results at 1.6 mGy with IR were neither confirmed at higher doses nor by the LROC analysis, and no significant dose effects were observed with DLR. In contrast, prior studies of IR and DLR in uniform phantoms reported dose-dependent results [11, 25–27]. This discrepancy can be explained by the different experimental setups we chose to more realistically reflect the diagnostic assessment of patients.

Anatomical background structure influences detection tasks conducted by radiologists and can outweigh the impact of quantum noise, ultimately limiting lesion perception [28]. Complex phantom structures were previously found to mitigate dose effects compared with simple uniform structures and to affect conclusions drawn regarding dose and image reconstruction [8, 9]. Our study aimed to investigate whether the advantages of DLR observed in uniform phantoms could be reproduced in a setting that better reflects clinical imaging. While our results confirm the superior performance of DLR, they show only moderate dose effects, which is due to the greater background complexity of the phantoms used in our study. These observations align with studies conducted on patients, which report minimal effects on the detection of similar-sized liver lesions within patient anatomy despite drastic dose reduction [10, 11].

We conducted separate ROC and LROC analyses to assess the effectiveness of DLR in enabling readers to determine lesion presence or absence (ROC) and to execute precise lesion delineation (LROC). Each analysis thus provided distinct insights into the image analysis performed by the readers and the utility of DLR for clinically relevant tasks. Our results demonstrated improvements in both aspects of image interpretation with DLR. The variations we observed in reader responses were caused by reader variability, a well-known factor in human observer studies [29]. This variability was more pronounced in the LROC analysis due to the inherently more complex task of precise lesion labeling compared to the ROC analysis.

Moreover, the level of experience also contributed to reader variability. We included a range of readers with different levels of experience to broaden our database for evaluating DLR. Training, knowledge, and experience play significant roles in influencing reader responses in clinical cancer trials [30–32]. In such trials, however, readers were tasked with accurately interpreting a variety of malignant image features, whereas our experiments focused solely on a specific detection task. Participants received precise instructions regarding the task and underwent a training session to become acquainted with the experimental setup. This explains why, despite slightly lower detection among less experienced readers, we found no significant difference in detection performance between reader groups.

DLR has been reported to improve image texture, accelerate reconstruction, and enable dose reduction in abdominal imaging [5, 10, 33]. Our study adds to these reports and confirms that DLR offers advantages when used in neck imaging. Nonetheless, it should be noted that DLR is a cover term for a family of algorithms that are based on different training data, intended for different applications, and may exhibit protocol-dependent performance [34]. Furthermore, despite the absence of significant dose effects in our experiments, DLR-induced dose reduction may compromise the conspicuity of very small low-contrast features and their characterization [10, 11]. We propose the use of realistic reference phantoms for diagnostic tasks to expand the evaluation of DLR, aiming for standardized assessment and ensuring the translatability of results to clinical imaging.

Our study has limitations. First, while we conducted our study using realistic anthropomorphic phantoms to simulate patients, we did not assess lesion detectability in real patients. Second, our results apply to the detection of low-contrast lesions that were selected to represent challenging and clinically relevant tasks. However, we cannot conclude on the detection of smaller or larger lesions or lesion classification. Third, we selected the same anatomical background for all experiments to ensure comparability, but detection results of the same lesion type may differ in different anatomical backgrounds. Fourth, we used phantom images acquired in a single CT scanner and we cannot provide evidence for DLR implementations of other vendors.

In conclusion, deep-learning reconstruction improves the detection of 1-cm low-contrast lesions in neck imaging compared with IR and filtered back projection, offering improved diagnostic performance and potential for dose reduction. Doses as low as 0.5 mGy may be used, if uncertainties related to the detectability of smaller features and their characterization are accepted.

## Data Availability

The primary data can be accessed under the following link: https://drive.google.com/drive/folders/1RhbEoTRjU73VyOVzEeDphActBOhPDA5q?usp=sharing

## Abbreviations

AiCE: Advanced intelligent Clear-IQ Engine
AIDR 3D: Adaptive iterative dose reduction three-dimensional
AUC: Area under the curve
CT: Computed tomography
DLR: Deep learning reconstruction
DSC: Dice similarity coefficient
FBP: Filtered back projection
IR: Iterative reconstruction
LROC: Localization receiver operating characteristic
ROC: Receiver operating characteristic
ROI: Region of interest
SEM: Standard error of the mean

## References

[1] Koetzier LR, Mastrodicasa D, Szczykutowicz TP et al (2023) Deep learning image reconstruction for CT: technical principles and clinical prospects. Radiology 306:e221257. 10.1148/radiol.221257 36719287 10.1148/radiol.221257 PMC9968777

[2] Solomon J, Marin D, Roy Choudhury K, Patel B, Samei E (2017) Effect of radiation dose reduction and reconstruction algorithm on image noise, contrast, resolution, and detectability of subtle hypoattenuating liver lesions at multidetector CT: filtered back projection versus a commercial model-based iterative reconstruction algorithm. Radiology 284:777–787. 10.1148/radiol.2017161736 28170300 10.1148/radiol.2017161736 PMC5702911

[3] Mileto A, Guimaraes LS, McCollough CH, Fletcher JG, Yu L (2019) State of the art in abdominal CT: the limits of iterative reconstruction algorithms. Radiology 293:491–503. 10.1148/radiol.2019191422 31660806 10.1148/radiol.2019191422

[4] Akagi M, Nakamura Y, Higaki T et al (2019) Deep learning reconstruction improves image quality of abdominal ultra-high-resolution CT. Eur Radiol 29:6163–6171. 10.1007/s00330-019-06170-3 30976831 10.1007/s00330-019-06170-3

[5] Racine D, Becce F, Viry A et al (2020) Task-based characterization of a deep learning image reconstruction and comparison with filtered back-projection and a partial model-based iterative reconstruction in abdominal CT: a phantom study. Phys Med 76:28–37. 10.1016/j.ejmp.2020.06.004 32574999 10.1016/j.ejmp.2020.06.004

[6] Greffier J, Hamard A, Pereira F et al (2020) Image quality and dose reduction opportunity of deep learning image reconstruction algorithm for CT: a phantom study. Eur Radiol 30:3951–3959. 10.1007/s00330-020-06724-w 32100091 10.1007/s00330-020-06724-w

[7] Njolstad T, Jensen K, Dybwad A, Salvesen O, Andersen HK, Schulz A (2022) Low-contrast detectability and potential for radiation dose reduction using deep learning image reconstruction-a 20-reader study on a semi-anthropomorphic liver phantom. Eur J Radiol Open 9:100418. 10.1016/j.ejro.2022.100418 35391822 10.1016/j.ejro.2022.100418 PMC8980706

[8] Solomon J, Ba A, Bochud F, Samei E (2016) Comparison of low-contrast detectability between two CT reconstruction algorithms using voxel-based 3D printed textured phantoms. Med Phys 43:6497. 10.1118/1.4967478 27908164 10.1118/1.4967478

[9] Conzelmann J, Genske U, Emig A, Scheel M, Hamm B, Jahnke P (2022) Comparison of low-contrast detectability between uniform and anatomically realistic phantoms-influences on CT image quality assessment. Eur Radiol 32:1267–1275. 10.1007/s00330-021-08248-3 34476563 10.1007/s00330-021-08248-3 PMC8794946

[10] Jensen CT, Gupta S, Saleh MM et al (2022) Reduced-dose deep learning reconstruction for abdominal CT of liver metastases. Radiology 303:90–98. 10.1148/radiol.211838 35014900 10.1148/radiol.211838 PMC8962777

[11] Lyu P, Liu N, Harrawood B et al (2023) Is it possible to use low-dose deep learning reconstruction for the detection of liver metastases on CT routinely? Eur Radiol 33:1629–1640. 10.1007/s00330-022-09206-3 36323984 10.1007/s00330-022-09206-3

[12] Ardila Pardo GL, Conzelmann J, Genske U, Hamm B, Scheel M, Jahnke P (2020) 3D printing of anatomically realistic phantoms with detection tasks to assess the diagnostic performance of CT images. Eur Radiol 30:4557–4563. 10.1007/s00330-020-06808-7 32221686 10.1007/s00330-020-06808-7 PMC7338819

[13] Jahnke P, Conzelmann J, Genske U et al (2021) Task-based assessment of neck CT protocols using patient-mimicking phantoms-effects of protocol parameters on dose and diagnostic performance. Eur Radiol 31:3177–3186. 10.1007/s00330-020-07374-8 33151393 10.1007/s00330-020-07374-8 PMC8043932

[14] Jahnke P, Limberg FR, Gerbl A et al (2017) Radiopaque three-dimensional printing: a method to create realistic CT phantoms. Radiology 282:569–575. 10.1148/radiol.2016152710 27626676 10.1148/radiol.2016152710

[15] Jahnke P, Schwarz S, Ziegert M, Schwarz FB, Hamm B, Scheel M (2019) Paper-based 3D printing of anthropomorphic CT phantoms: feasibility of two construction techniques. Eur Radiol 29:1384–1390. 10.1007/s00330-018-5654-1 30116957 10.1007/s00330-018-5654-1

[16] Genske U, Jahnke P (2022) Human Observer Net: a platform tool for human observer studies of image data. Radiology 303:524–530. 10.1148/radiol.211832 35258375 10.1148/radiol.211832

[17] Chakraborty D (2018) Observer performance methods for diagnostic imaging: foundations, modeling, and applications with R-based examples. CRC Press, Boca Raton

[18] Chakraborty D, Zhai X (2023) RJafroc: artificial intelligence systems and observer performance. R package version 2.1.3., Available via https://dpc10ster.github.io/RJafroc/

[19] Dice LR (1945) Measures of the amount of ecologic association between species. Ecology 26:297–302. 10.2307/1932409 10.2307/1932409

[20] Sørensen T (1948) A method of establishing groups of equal amplitude in plant sociology based on similarity of species and its application to analyses of the vegetation on Danish commons. Biologiske Skrifter/Kongelige Danske Videnskabernes Selskab 5:1–34

[21] Joemai RM, Veldkamp WJ, Kroft LJ, Hernandez-Giron I, Geleijns J (2013) Adaptive iterative dose reduction 3D versus filtered back projection in CT: evaluation of image quality. AJR Am J Roentgenol 201:1291–1297. 10.2214/AJR.12.9780 24261369 10.2214/AJR.12.9780

[22] Goenka AH, Herts BR, Obuchowski NA et al (2014) Effect of reduced radiation exposure and iterative reconstruction on detection of low-contrast low-attenuation lesions in an anthropomorphic liver phantom: an 18-reader study. Radiology 272:154–163. 10.1148/radiol.14131928 24620913 10.1148/radiol.14131928

[23] Mieville FA, Gudinchet F, Brunelle F, Bochud FO, Verdun FR (2013) Iterative reconstruction methods in two different MDCT scanners: physical metrics and 4-alternative forced-choice detectability experiments-a phantom approach. Phys Med 29:99–110. 10.1016/j.ejmp.2011.12.004 22217444 10.1016/j.ejmp.2011.12.004

[24] Urikura A, Ichikawa K, Hara T, Nishimaru E, Nakaya Y (2014) Spatial resolution measurement for iterative reconstruction by use of image-averaging techniques in computed tomography. Radiol Phys Technol 7:358–366. 10.1007/s12194-014-0273-2 24880960 10.1007/s12194-014-0273-2

[25] Schindera ST, Odedra D, Raza SA et al (2013) Iterative reconstruction algorithm for CT: can radiation dose be decreased while low-contrast detectability is preserved? Radiology 269:511–518. 10.1148/radiol.13122349 23788715 10.1148/radiol.13122349

[26] McCollough CH, Yu L, Kofler JM et al (2015) Degradation of CT low-contrast spatial resolution due to the use of iterative reconstruction and reduced dose levels. Radiology 276:499–506. 10.1148/radiol.15142047 25811326 10.1148/radiol.15142047 PMC4514568

[27] Greffier J, Dabli D, Frandon J et al (2021) Comparison of two versions of a deep learning image reconstruction algorithm on CT image quality and dose reduction: a phantom study. Med Phys 48:5743–5755. 10.1002/mp.15180 34418110 10.1002/mp.15180

[28] Bochud FO, Valley JF, Verdun FR, Hessler C, Schnyder P (1999) Estimation of the noisy component of anatomical backgrounds. Med Phys 26:1365–1370. 10.1118/1.598632 10435539 10.1118/1.598632

[29] Garland LH (1959) Studies on the accuracy of diagnostic procedures. Am J Roentgenol Radium Ther Nucl Med 82:25–38 13661490

[30] Lee HJ, Goo JM, Lee CH et al (2009) Predictive CT findings of malignancy in ground-glass nodules on thin-section chest CT: the effects on radiologist performance. Eur Radiol 19:552–560. 10.1007/s00330-008-1188-2 18925404 10.1007/s00330-008-1188-2

[31] Miglioretti DL, Gard CC, Carney PA et al (2009) When radiologists perform best: the learning curve in screening mammogram interpretation. Radiology 253:632–640. 10.1148/radiol.2533090070 19789234 10.1148/radiol.2533090070 PMC2786195

[32] Schmid AM, Raunig DL, Miller CG et al (2021) Radiologists and clinical trials: part 1 the truth about reader disagreements. Ther Innov Regul Sci 55:1111–1121. 10.1007/s43441-021-00316-6 34228319 10.1007/s43441-021-00316-6 PMC8259547

[33] Bornet PA, Villani N, Gillet R et al (2022) Clinical acceptance of deep learning reconstruction for abdominal CT imaging: objective and subjective image quality and low-contrast detectability assessment. Eur Radiol 32:3161–3172. 10.1007/s00330-021-08410-x 34989850 10.1007/s00330-021-08410-x

[34] Yang K, Cao J, Pisuchpen N et al (2023) CT image quality evaluation in the age of deep learning: trade-off between functionality and fidelity. Eur Radiol 33:2439–2449. 10.1007/s00330-022-09233-0 36350391 10.1007/s00330-022-09233-0

